# Transverse colon cancer occurring at a colostomy site 35 years after colostomy: a case report

**DOI:** 10.1186/s12957-015-0593-7

**Published:** 2015-05-06

**Authors:** Chiyo Maeda, Eiji Hidaka, Mari Shimada, Shoji Shimada, Kenta Nakahara, Daisuke Takayanagi, Yusuke Takehara, Shumpei Mukai, Naruhiko Sawada, Fumio Ishida, Shin-ei Kudo

**Affiliations:** Digestive Disease Center, Showa University Northern Yokohama Hospital, 35-1, Chigasaki-Chuo, Tsuzuki-ku, Yokohama, 224-8503 Japan

**Keywords:** Colostomy site, Ostomate, Colorectal cancer

## Abstract

**Background:**

Carcinomas occurring at colostomy sites are rare, and most of these are metachronous colorectal cancers. The median time between colostomy and development of a carcinoma at a colostomy site is 22 years, which exceeds the length of the recommended follow-up period. We report a rare case of a carcinoma of the transverse colon occurring at a colostomy site in a patient without a history of colorectal cancer.

**Case report:**

An 89-year-old woman presented with a tumor occurring at a colostomy site. Thirty-five years previously, she had undergone a transverse loop colostomy for an iatrogenic colon perforation that occurred during left ureteral lithotomy. Upon physical examination, the patient had a hard nodule measuring 3 cm at the colostomy site. A biopsy of the nodule suggested adenocarcinoma, and the preoperative diagnosis was transverse colon cancer. A laparotomy was performed via a peristomal incision with 5-mm skin margins, and the tumor was covered by a surgical glove to avoid any tumor seeding. The colon was separated from the tumor by 5-cm margins, and the specimen was removed *en bloc*. An end colostomy was constructed to a new site on the right side of the abdomen. The deficit in the abdominal wall was repaired, and the skin was closed via a purse-string suture. The final diagnosis of the stoma tumor was transverse colon cancer (T2, N0, M0, stage I). One year and five months after surgery, there was no evidence of recurrence.

**Conclusions:**

The occurrence of carcinomas at colostomy sites in patients without a history of colorectal cancer is rare. It is important to train ostomates to monitor the stoma for possible tumor recurrence.

## Background

Carcinomas occurring at colostomy sites are rare, with only 12 cases previously reported in the English literature (Table 1); most of these are metachronous colorectal cancers. Cases without malignant potential, such as those involving colorectal cancer or ulcerative colitis, are very rare. Herein, we describe a case of an 89-year-old woman who presented with a colostomy tumor 35 years after undergoing a transverse colostomy for iatrogenic colon perforation, and we review the literature related to this malignancy.

**Table 1:** **Twelve previously reported cases of carcinomas at a stoma site**

**Author**	**Year**	**Age/sex**	**Past history**	**Term (years)**	**Chief complaint**	**Size (cm)**	**Depth**	**Histology**	**Prognosis**
Morgan	1966	76/F	Ulcerative colitis	31 years after colostomy	Bleeding	7.4 × 6.4	SI (skin)	Adenocarcinoma	-
Didolkar	1975	42/F	Rectal carcinoma	32 years after colostomy	Stoma ulcer	3 to 4	-	Basal cell carcinoma	Alive (2.5 years)
Takami	1983	53/M	Rectal carcinoma	19 years after APR	Tumor	16	SI (skin)	Mod, muc	Alive (2 years)
Kusunoki	1996	63/M	Ulcerative colitis	6 years after colostomy	-	-	MP	Mod, muc	Alive (5 years)
Shibuya	2002	57/M	Rectal carcinoma	8 years after APR	Stoma stenosis	6 × 4	SI (skin)	Mod	Alive (4 years)
Townley	2005	57/F	Rectal carcinoma	5 years after APR	Tumor	3	Dukes A	Adenocarcinoma	Alive (6 months)
Papaziogas	2006	77/F	Rectal carcinoma	3 months after Hartmann	Tumor	2.5	-	Adenocarcinoma	Death (1 year)
Chintamani	2007	30/M	Rectal carcinoma	6 years after APR	Stoma stenosis	-	T4N1	Por, sig	Death (4 years)
Vijayasekar	2008	61/F	Rectal carcinoma	14 years after APR	Tumor	-	SI (subcutaneous)	Well	-
Okamoto	2009	67/M	Rectal carcinoma	15 years after APR	Tumor	8 × 10.5	SI (muscle)	Well	-
Sabater-Marco	2013	61/M	Lung/rectal carcinoma	6 years after APR	Tumor	-	-	Large cell carcinoma	Death (3 months)
Maurra	2014	75/F	Sigmoid volvulus	50 years after Hartmann	Tumor	-	SI (muscle)	Well, muc	Alive (8 years)
Our case	2014	89/F	Iatrogenic perforation	35 years after colostomy	Bleeding	3.3	MP	Mod	Alive (11 months)

F: female, M: male, APR: abdominoperineal resection, mod: moderately differentiated adenocarcinoma, muc: mucinous adenocarcinoma, SI: infiltration to other organ, MP: proper muscle.

## Case presentation

**Figure 1:**
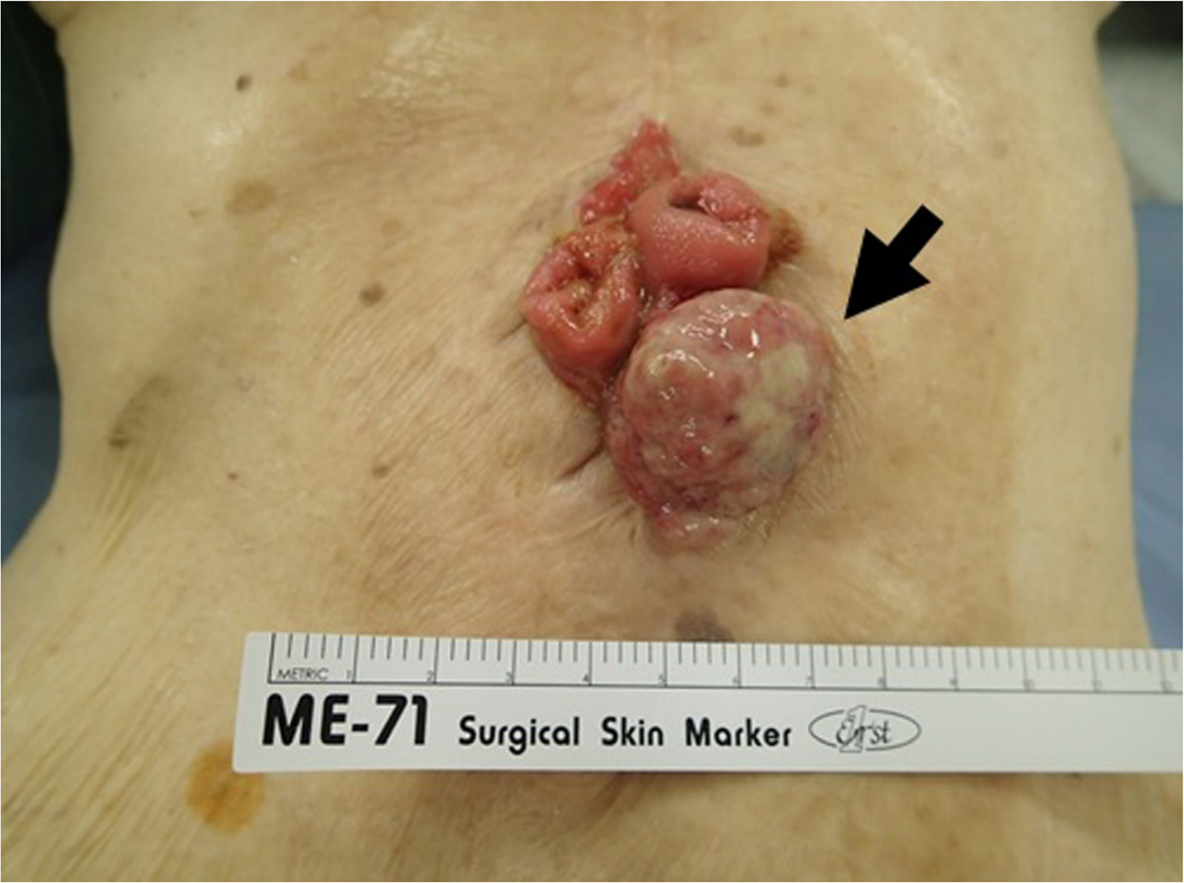
Macroscopic findings on admission. The patient had a hard nodule that measured 3 cm at the colostomy site. The arrows indicate the tumor.

An 89-year-old Japanese woman with a 1-month history of stoma bleeding was referred to our hospital. She had undergone a transverse loop colostomy 35 years previously for an iatrogenic colon perforation that occurred during left ureteral lithotomy. Upon physical examination, the patient was found to have a hard nodule at the colostomy site, measuring 3 cm (Figure 1). A biopsy of the nodule suggested adenocarcinoma. Laboratory data revealed anemia and renal dysfunction. Serum carcinoembryonic antigen and carbohydrate antigen 19-9 levels were not elevated. Staging computed tomography showed a 3-cm tumor adjacent to the stoma with no invasion into the abdominal muscle. There was no evidence of metastatic disease. Colonoscopy via the stoma revealed no other lesions in the colon or rectum. On the basis of our findings, the preoperative diagnosis of the stoma tumor was transverse colon cancer (T2, N0, M0, stage I). We concluded that colectomy and repositioning of the colostomy site were appropriate for this patient. Although we thought stoma closure was possible, the patient and her family were opposed to this strategy because they were concerned about anal function.

**Figure 2:**
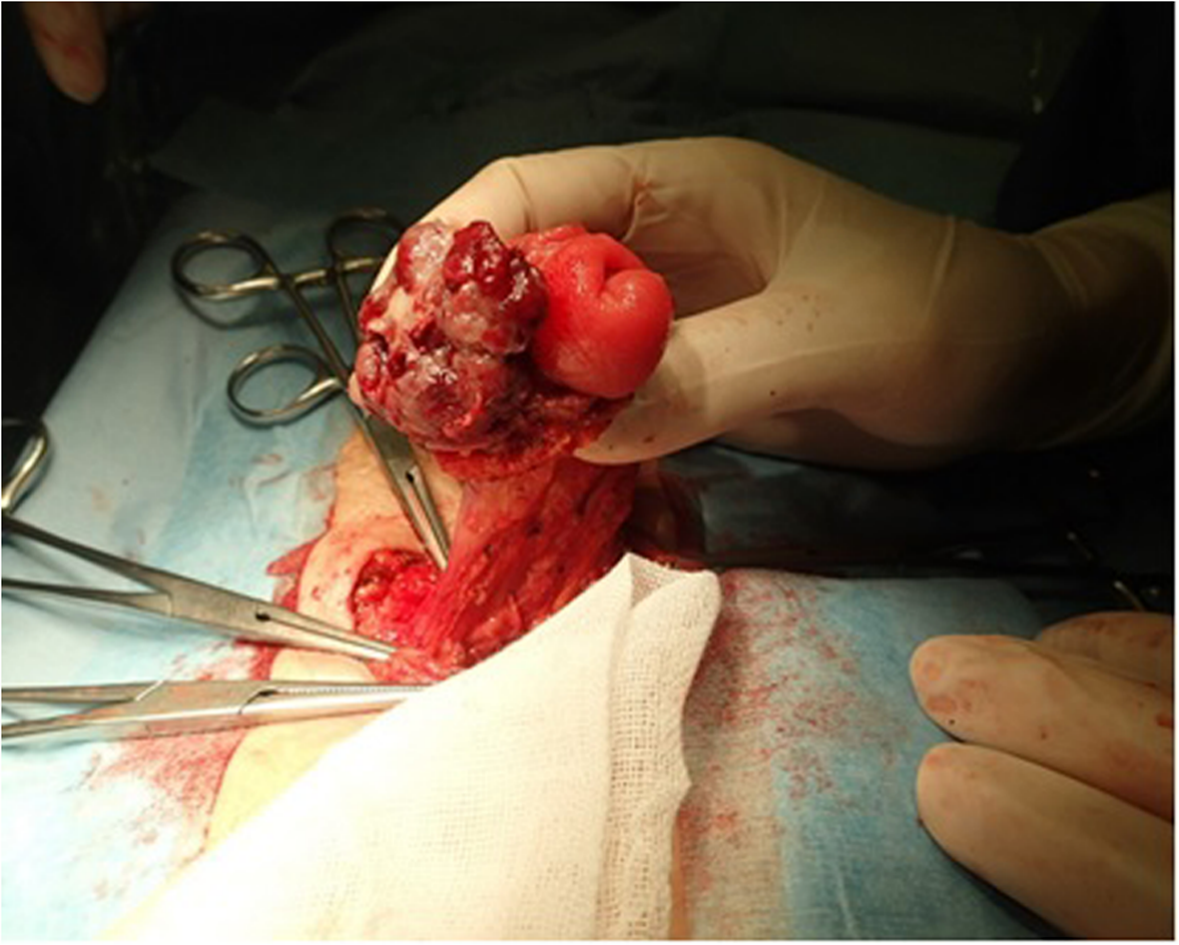
Surgical findings. The tumor was removed *en bloc*.

**Figure 3:**
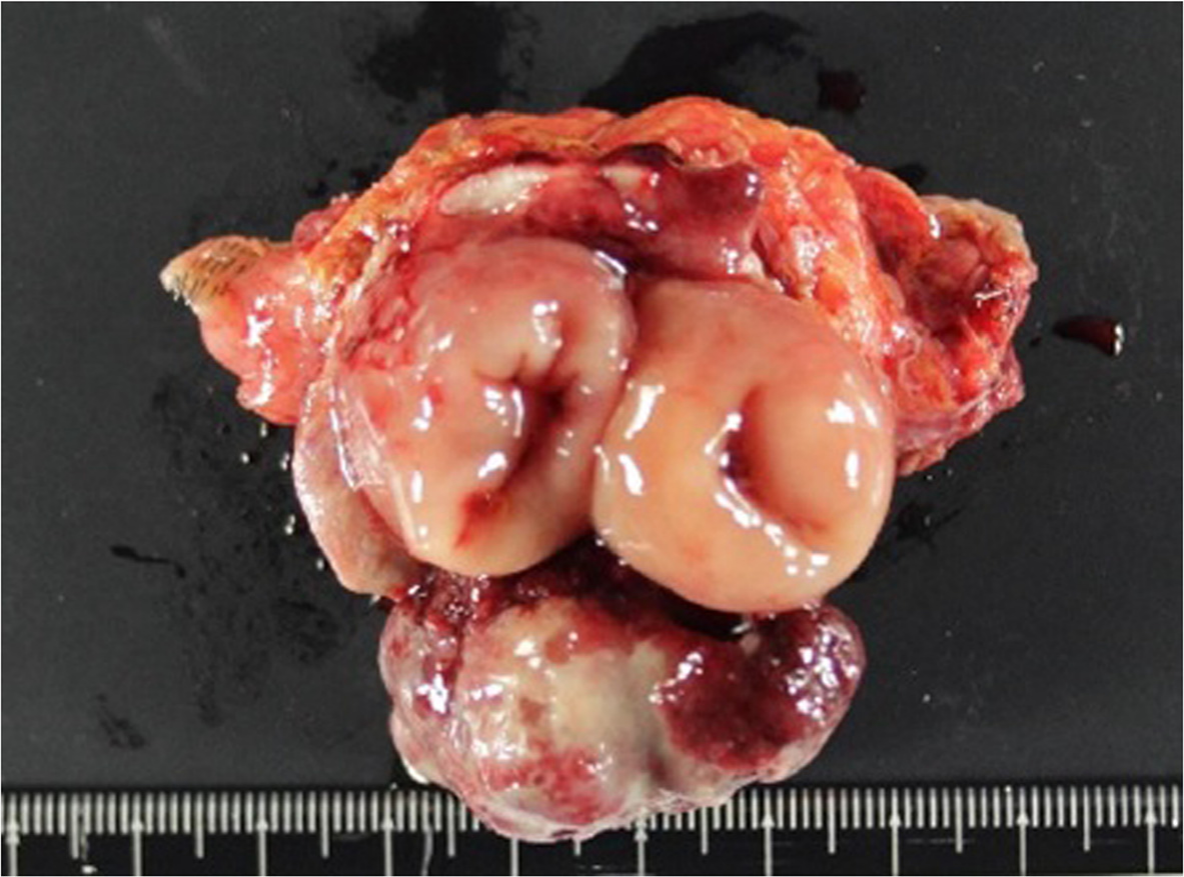
Macroscopic findings. Macroscopic examination showed complete excision of the tumor with clear margins.

Under general anesthesia, a laparotomy was performed via a peristomal incision with 5-mm skin margins, and the tumor was covered using a surgical glove to avoid tumor seeding. Adhesion around the stoma was not strong, and there was no evidence of peritoneal dissemination. Only pericolic lymph nodes were dissected. The colon was separated from the tumor by 5-cm margins, and the specimen was removed *en bloc* (Figure 2). An end colostomy was constructed to a new site on the right side of the abdomen. The deficit in the abdominal wall was repaired, and the skin was closed via a purse-string suture. The operative time was 102 min, and blood loss was 52 mL. Macroscopic examination showed complete excision of the tumor with clear margins (Figure 3).

The patient was discharged 8 days after surgery. Her postoperative course was uneventful, and she recovered without any complications. One year and five months after surgery, there is no evidence of recurrence.

**Figure 4:**
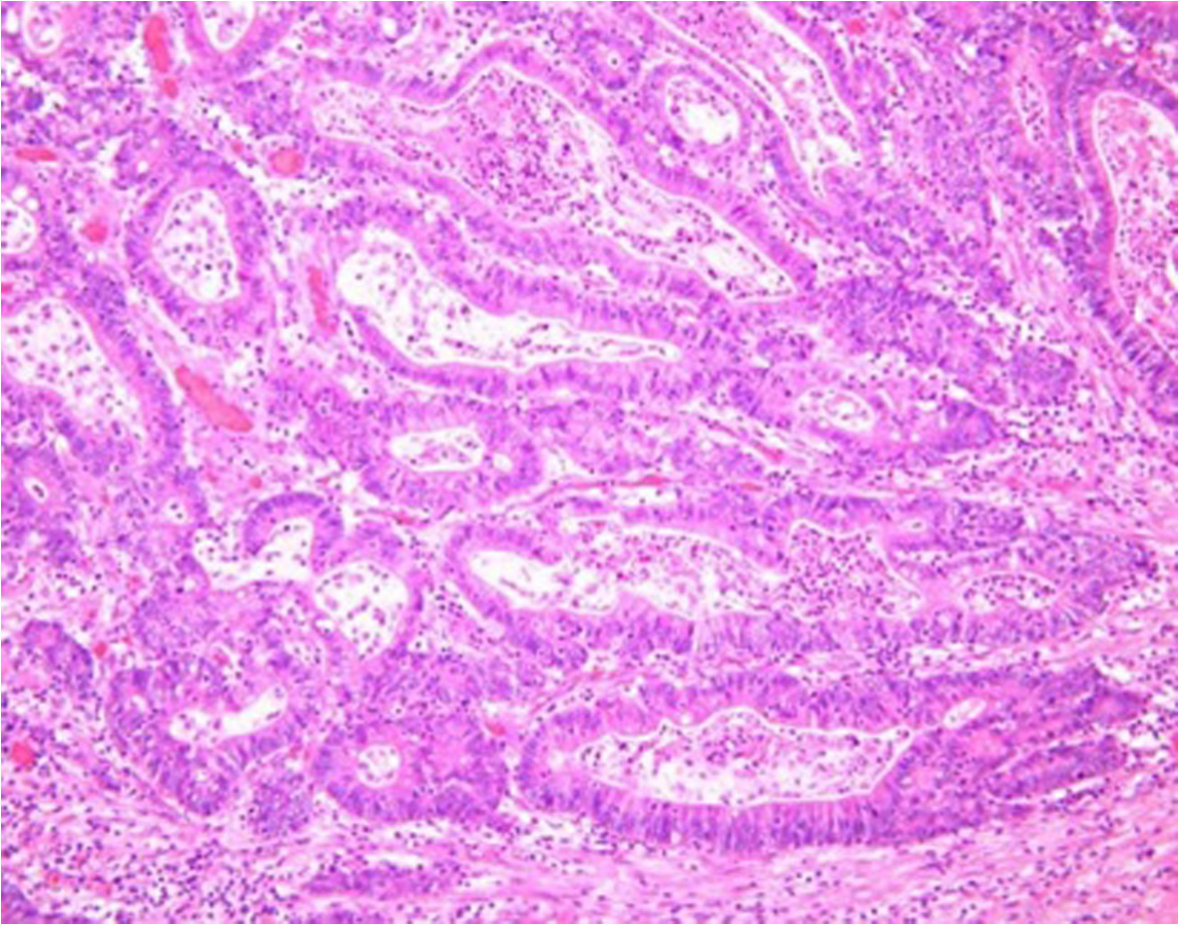
Microscopic findings. Microscopic examination showed moderately differentiated adenocarcinoma mixed with well-differentiated adenocarcinoma.

Microscopic examination of the tumor showed moderately differentiated adenocarcinoma mixed with well-differentiated adenocarcinoma (Figure 4). The tumor extended into the proper muscle layer and directly invaded the skin. There was no lymph node metastasis. The final diagnosis of the stoma tumor was transverse colon cancer (T2, N0, M0, stage I).

The study protocol was approved by the ethics committee of Showa University Northern Yokohama Hospital. The study was conducted according to the Declaration of Helsinki.

## Discussion

Primary adenocarcinoma arising at a colostomy site is rare. The first case was reported by Morgan in 1966 [1] and involved a patient whose underlying disease was ulcerative colitis. The 12 cases reported previously involved patients with metachronous colon cancer (eight patients), ulcerative colitis (two patients), metachronous lung cancer (one patient) [2], and a sigmoid volvulus (one patient) [3] (Table 1). Malignant potential was lacking only in the sigmoid volvulus case and in the case reported here. Cancers arising near stomas typically show skin metastasis [4] and include metachronous cancers. Immunopathological staining of cytokeratin facilitates the differential diagnosis [2].

Cutaneous metastasis of colon cancers to operation scars after cancer resection accounts for 0.6% of cases of recurrence [5]. The mean interval to the development of skin metastasis after colon cancer diagnosis is 4.9 years [6]. The average survival of patients with skin metastasis is 7.5 months after diagnosis [7], whereas the median time to the development of a carcinoma at a colostomy site after the initial operation is 22 years [8]. Most stoma site carcinomas are diagnosed as advanced cancers. The Japanese national guidelines [9] recommend a 5-year follow-up period after colorectal cancer resection. Thereafter, self-observation of the stoma by the patient is very important for the early detection of cancer. In our hospital, nurses who specialize in stoma care train ostomates in the methods of self-care and self-observation at the outpatient clinic for several years. The patient in the case reported here had not received this training and did not know about the possibility of a carcinoma arising at the stoma site. It was also difficult for her to recognize the tumor because it grew slowly over several years. Thus, it is important to train ostomates in regard to self-observation of the stoma.

Of the 12 previously reported cases of adenocarcinomas arising at colostomies, six involved patients with T4 colon cancer that invaded the skin or subcutaneous tissue (pkpk). In the literature, it is not clear whether tumors directly invade the skin or penetrate the colon serosa. In the patient described in this report, tumor invasion included the proper muscle layer, with direct invasion of the skin. The depth of colostomy site cancer needs to be clarified in terms of direct invasion or invasion through the colon serosa.

Our patient had no history of cancer, radiation therapy, or ulcerative colitis; the case reported here therefore represents an extremely rare case of colostomy site carcinoma that did not have malignant potential. The etiology in this case is unclear. The carcinoma may have resulted from physical stimulation caused by clothing or unexpected compression, or by chemical stimulation, such as that resulting from enterobacteria or bile acids in stools [10]. On the other hand, stoma site cancers could also occur coincidentally. The incidence of metachronous colorectal cancer after abdominoperineal resection for rectal cancer is 2.2% [11]. Most metachronous tumors (51%) are located in the left hemicolon, as are most stomas after colorectal cancer. Therefore, in this case, it seems that a colon cancer occurred coincidentally at the stoma site; however, physical stimulation or chemical stimulation could promote their occurrence.

In surgical operations, it is best not to expose the tumor. Cutaneous recurrence at the stoma closure suture after 5 years has been reported [12]. In this case, the tumor was not exposed because it was covered by a surgical glove. Because surgical site infection is very common after stoma closure, a purse-string skin closure [13], which reduces wound infection [14], was performed in this case.

The number of reports describing ileostomy site carcinoma is greater than the number of reports describing colostomy site carcinoma [15]. *En bloc* resection of the ileostomy, wide resection of the adjacent anterior abdominal wall, and transposition of the stoma to a new site have been shown to provide the best prognosis for a patient diagnosed with adenocarcinoma following ileostomy [16].

The mechanism of cancers arising at ileostomy sites is also speculative. Most grow at the mucocutaneous junction [17]. Previous reports have proposed that physical trauma or chemical or physical irritation predisposes the ileal mucosa to colonic metaplasia, dysplasia, and malignant change. Although there are many more colorectal cancer patients than small intestine cancer patients, there are fewer case reports of colostomy site carcinomas than ileostomy site carcinomas. The mechanism of cancer development at ileostomy sites differs from that at colostomy sites. Chronic metaplasia and dysplasia were found in the ileal mucosa adjacent to tumors in patients with ulcerative colitis and familial adenomatous polyposis [15], suggesting that the malignant potential might be high in patients with these underlying diseases.

## Conclusions

Primary adenocarcinomas arising at colostomy sites are rare. Colostomy site carcinomas may occur after the postoperative follow-up period has ended. In order to facilitate early detection, it is important to train ostomates to monitor the stoma for possible tumors.

## Consent

Written informed consent for the publication of this case report and any accompanying images was obtained from the patient’s family. A copy of the written consent is available for review by the Editor-in-Chief of this journal.
